# Evidence of New Endemic Clusters of Human T-Cell Leukemia Virus (HTLV) Infection in Bahia, Brazil

**DOI:** 10.3389/fmicb.2019.01002

**Published:** 2019-05-14

**Authors:** Felicidade Mota Pereira, Maria da Conceição Chagas de Almeida, Fred Luciano Neves Santos, Roberto Perez Carreiro, Carlos Gustavo Regis-Silva, Bernardo Galvão-Castro, Maria Fernanda Rios Grassi

**Affiliations:** ^1^Laboratório Avançado de Saúde Pública, Fundação Oswaldo Cruz, Salvador, Brazil; ^2^Laboratório Central de Saúde Pública Prof. Gonçalo Moniz – Secretaria da Saúde do Estado da Bahia, Salvador, Brazil; ^3^Laboratório de Epidemiologia Molecular e Bioestatística, Fundação Oswaldo Cruz, Salvador, Brazil; ^4^Centro de Integração de Dados e Conhecimentos para Saúde, Fundação Oswaldo Cruz, Salvador, Brazil; ^5^Escola Bahiana de Medicina e Saúde Pública, Salvador, Brazil

**Keywords:** HTLV, clusters, epidemiology, Bahia, spatial distribution

## Abstract

**Background:**

Salvador, Bahia (northeastern Brazil), has been identified as the epicenter of Human T-cell leukemia virus Human T-cell leukemia virus (HTLV) type 1 infection in the country. This study aims to estimate the rate of HTLV infection and the geographical distribution of this virus in this state.

**Methods:**

All HTLV tests (chemiluminescence/ELISA assays/Western Blotting) performed in the Central Laboratory of Public Health of Bahia (LACEN) from 2004 to 2013 were included. Data was extracted from LACEN’s database using high volume extract, transformation and load throughput. Infection rate was expressed as the number of infected individuals per 100,000 inhabitants considering municipalities grouped in microregions and/or mesoregions as the unit of analysis.

**Results:**

A total of 233,876 individuals were evaluated. Individuals were from 394 out of 417 municipalities of Bahia (94.5%). HTLV chemiluminescence/ELISA assay was found to be reactive for 3,138 individuals from whom 2,323 had WB results (1,978 positives, 62 negative and 282 indeterminate). Out of 1978 reactive samples, 1,813 (91.7%) were positive for HTLV-1, 58 (2.9%) for HTLV-2 and 107 (5.4%) were for both HTLV-1 and HTLV-2. The cumulative mean rate of HTLV-positive cases in Bahia was 14.4 per 100,000 inhabitants. Three microregions presented rates >20 HTLV-positive cases/100,000 inhabitants: Barreiras (24.83 cases per 100,000 inhabitants), Salvador (22.90 cases per 100,000 inhabitants), and Ilhéus-Itabuna (22.60 cases per 100,000 inhabitants).

**Conclusion:**

HTLV infection is disseminated in the state of Bahia, with an overall moderate rate of infection. Further studies should be conducted to characterize the epidemiological and clinical profile of HTLV-infected individuals better and to propose effective prevention measures.

## Introduction

Human T-cell leukemia virus (HTLV) type 1 was the first human oncogenic retrovirus to be identified in 1979 (Poiesz et al., 1980). Subsequently, in 1982, HTLV-2 was identified (Kalyanaraman et al., 1982). HTLV types 3 and 4 were identified only in 2005 in samples of patients from Cameroon (Calattini et al., 2005; Wolfe et al., 2005). To date, only HTLV-1 has been associated with the development of severe diseases, such as adult T-cell leukemia/lymphoma (Hinuma et al., 1981) and tropical spastic paraparesis/HTLV-1 associated myelopathy (TSP/HAM) (Gessain et al., 1985; Osame et al., 1986), uveitis (Mochizuki et al., 1992) and infective dermatitis (LaGrenade et al., 1990). HTLV-1 infection has also been associated with inflammatory diseases, such as bronchiectasis (Einsiedel et al., 2014; Honarbakhsh and Taylor, 2015), Keratoconjunctivitis sicca (Castro-Lima Vargens et al., 2011) and arthritis (Yakova et al., 2005). In addition, HTLV-1-infected individuals are known to be more susceptible to infectious diseases, such as tuberculosis (Marinho et al., 2005; Verdonck et al., 2007a; Grassi et al., 2016), disseminated *Strongyloides stercoralis* (Nera et al., 1989) and Norwegian scabies (Brites et al., 2002). The transmission of HTLV-1 occurs through the transfusion of contaminated blood or tissue, including needle sharing among drug users, from mother-to-child, predominantly through breastfeeding (Bittencourt et al., 2001), as well as through sexual contact (Gessain and Cassar, 2012). Recently, the sexual transmission of HTLV-1 was reported as the main route in Salvador, Bahia (Nunes et al., 2017).

It is estimated that at least 5–10 million people are infected with HTLV-1 worldwide, with the highest prevalence occurring in Japan, on the African continent, in the Caribbean, the Melanesian Islands and South America (Gessain and Cassar, 2012). HTLV-2 is less prevalent and is predominantly found in the indigenous populations of North, Central, and South America, as well as in injection drug users in the United States and Europe (Ishak et al., 1995; Hall et al., 1996).

Brazil is home to around 800,000 HTLV-1-infected people and represents one of the largest endemic areas for the diseases associated with HTLV-1 (Gessain and Cassar, 2012). The virus is present throughout the country, with variable prevalence in accordance with geographical region, being highest in the Northeast and North regions. Salvador, the capital of the state of Bahia, has been identified as the epicenter of HTLV-1 infection in Brazil (Galvao-Castro et al., 1997; Catalan-Soares et al., 2005). A population-based study conducted in this city found that 1.76% of the population was infected with HTLV-1. A higher prevalence was found among women, reaching 10% of those aged 50 years or older (Dourado et al., 2003).

Bahia is the largest state in the Brazilian Northeast, with a total of 417 municipalities. Sparse studies have indicated that HTLV-1 infection may be present in some regions of the state. However, these studies have investigated specific population groups, such as pregnant women, blood donors or drug users (Dourado et al., 1998; Magalhaes et al., 2008; Boa-Sorte et al., 2014; Mello et al., 2014). Large-scale population-based assessments are considered the gold standard to determine the prevalence of a given infection; however, the high cost associated with these make them difficult to perform (Hlela et al., 2009). The present study aimed to estimate the seroprevalence and geographical distribution of HTLV infection in the state of Bahia over a 10-year period (2004–2013). All available serological tests for HTLV- 1/2 were evaluated using the database of the Bahia state public health reference laboratory, which tests blood samples from all municipalities in the state.

## Materials and Methods

### Ethics Statement

The Institutional Review Board (IRB) for Human Research at the Gonçalo Moniz Institute of the Oswaldo Cruz Foundation (Salvador, Bahia, Brazil) provided ethical approval to conduct this study (CAAE number 22478813.7.0000.0040).

### Study Area

**FIGURE 1:**
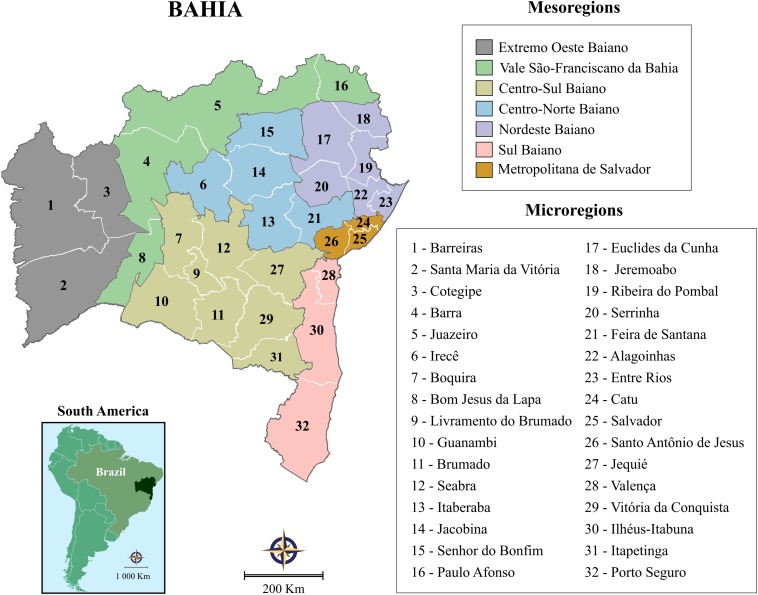
Illustration of the state of Bahia’s seven mesoregions and 32 microregions, grouped by IBGE to more accurately reflect economic and social similarities among this state’s municipalities.

This study was performed in the state of Bahia, Brazil, the fourth largest state in terms of population size, and the fifth largest in terms of area: 565,733 km^2^. The state is comprised of 417 municipalities, which have been grouped into 32 microregions and seven mesoregions by the Brazilian National Institute of Geography and Statistics (IBGE) in accordance with the economic and social similarities among them (Figure 1). According to the 2015 Brazilian national census, the state’s total population size was 15,203,934 inhabitants, with an overall density of 27 inhabitants per km^2^ (http://www.ibge.gov.br).

### Study Design

A retrospective ecological study was conducted using data obtained from the Central Laboratory of Public Health of Bahia (LACEN-BA), which is responsible for infectious disease surveillance throughout the state via laboratory analysis. The target population was comprised mainly of blood donors, pregnant women and individuals exhibiting symptoms of infectious disease, referred by blood banks, prenatal physicians or clinicians in the public health system.

All serological tests for HTLV were included among the 32 Bahia microregions from 2004 to 2013. From 2004 to 2008, testing was carried out by ELISA using the Murex HTLV-1/2 immunoassay (DiaSorin S.p.A., Dartford, United Kingdom), from 2009 to 2010 using the anti-HTLV-1/2 Sym Solution kit (Symbiosis Diagnostica LTDA, Leme, Brazil) and, from 2011, the CLIA chemiluminescence assay (Architect rHTLV-1/2, Abbott Diagnostics Division, Wiesbaden, Germany) was used. When HTLV seropositivity was detected, Western blotting (HTLV Blot 2.4, Genelabs Diagnostics^®^, Singapore) was used throughout the study period for confirmation. HTLV-negative samples were defined as lack of reactivity to HTLV-specific proteins; HTLV-1-positive samples were defined as reactive to GAG (p19 with or without p24) and two ENV (GD21 and rgp46-I) proteins; HTLV-2-positive samples were those reactive to GAG (p24 with or without p19) and two ENV (GD21 and rgp46-II) proteins; HTLV seropositive samples were defined as samples reactive to GAG (p19 and p24) and ENV (GD21). The samples that indicated co-infection of HTLV -1/2 demonstrated reactivity for specific markers of both HTLV-I and HTLV-II: GD21, p19, p24, rgp46-II and rgp46-I. Samples were considered indeterminate when no specific bands for HTLV-I, HTLV-II, or HTLV were detected. All serological tests for HTLV that lacked confirmatory results were excluded.

### Data Analysis

All serological tests performed in each year of the study period were extracted from the SMART LAB laboratory management system using high volume extract, transformation and load throughput. Each individual’s unique registration number was considered as the key variable. In order to avoid multiplicity, each individual’s most recent serological result was considered. The database was validated using the R software package and analyzed with STATA v13.0. Median and interquartile range (IQR) intervals were calculated for the age variable, and individuals were grouped accordingly. Absolute and relative frequencies were calculated for all categorical variables (age groups: <15/15–30/31–50/>50; sex: male/female, and serological test results: reactive/non-reactive). Geographic information system techniques and spatial analysis tools were employed to determine the geographical distribution of HTLV throughout the state of Bahia. Population data were acquired from IBGE, supported by national census data from 2010 and official estimates for all other years^1^.

Infection rate was expressed as the number of infected individuals per 100,000 inhabitants. Microregions and/or mesoregions were adopted as the unit of analysis in order to obtain improved accuracy concerning differences among regions. Digital maps were obtained from the IBGE cartographic database in shapefile (.shp), which were subsequently reformatted and analyzed using TerraView version 4.2, open source software freely available from the National Institute for Space Research^2^. Spatial distribution maps based on moving averages were constructed by applying 3-year intervals to data from 2004 to 2013. This method was also used to minimize the effects of random fluctuation for a time-series of infection rates calculated for rare events. Annual incidence rates were calculated for each of the 32 micro-regions and the entire state using standardized methods. To assess the relative risk of HTLV in each microregion, maps were constructed using the moving average for the first 3-year rate in Bahia as the denominator assuming no changes in time and space.

## Results

**FIGURE 2:**
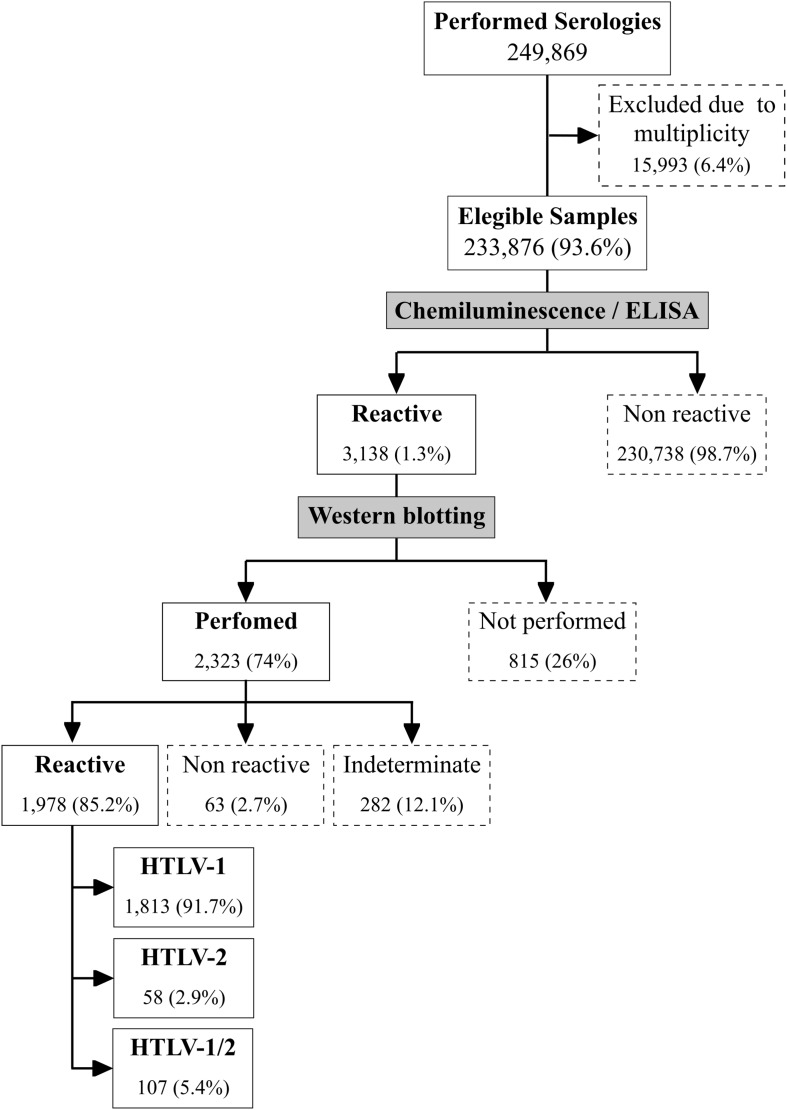
Standards for reporting of diagnostic accuracy studies (STARD) flowchart illustration of study design. Boxes with dotted lines indicate samples excluded from the study.

A total of 249,869 serologies for HTLV were performed during the studied period. Due to multiplicity (i.e., the presence of multiple serological test results in the database for a given individual), 15,993 samples were excluded (Figure 2). The final sample comprised 233,876 serological tests from unique individuals. Out of 3,138 HTLV-positive samples, the 2,323 samples submitted to Western blotting produced 1,978 positive (85.2%) 63 negative (2.7%) and 282 (12.1%) indeterminate results, yielding an overall prevalence of 0.84%. However, 815 samples were not submitted to Western Blot due to a lack of reagents at some points during the study period, and were excluded from the analysis. Assuming that 85.2% of these 815 samples Blot would have been HTLV positive, it follows that the overall prevalence of HTLV would have been 1.18%.

**FIGURE 3:**
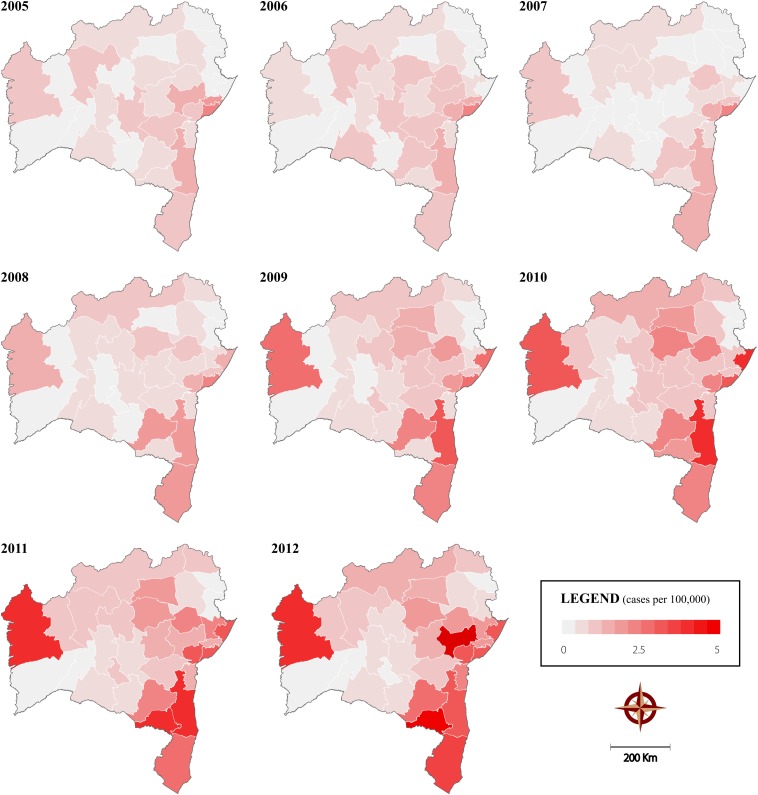
Spatiotemporal distribution of HTLV positivity in the State of Bahia from 2004 to 2013, calculated using 3-year moving averages considering the state’s microregions as units of analysis.

Of the positive samples, 1,813 (91.7%) were positive for HTLV-1 (prevalence of 0.78%), 58 (2.9%) for HTLV-2 (prevalence of 0.025%) and 107 (5.4%) were positive for both HTLV-1 and HTLV-2 (prevalence of 0.05%). A total of 394 (94.5%) of the 417 municipalities in the state reported at least one positive result during the study period. Information regarding the municipality of sample origin was missing for 1.4% of the HTLV-positive samples. Figure 3 illustrates the spatiotemporal distribution of HTLV positivity at eight distinct time points. An overall increase in the number of positive cases was observed in all microregions, most notably after 2008, as evidenced by the first 5 years of study (2004–2008) in which 4.3 cases of HTLV per 100,000 inhabitants was found, compared to 10.5 cases per 100,000 inhabitants during the final period (2009–2013).

**FIGURE 4:**
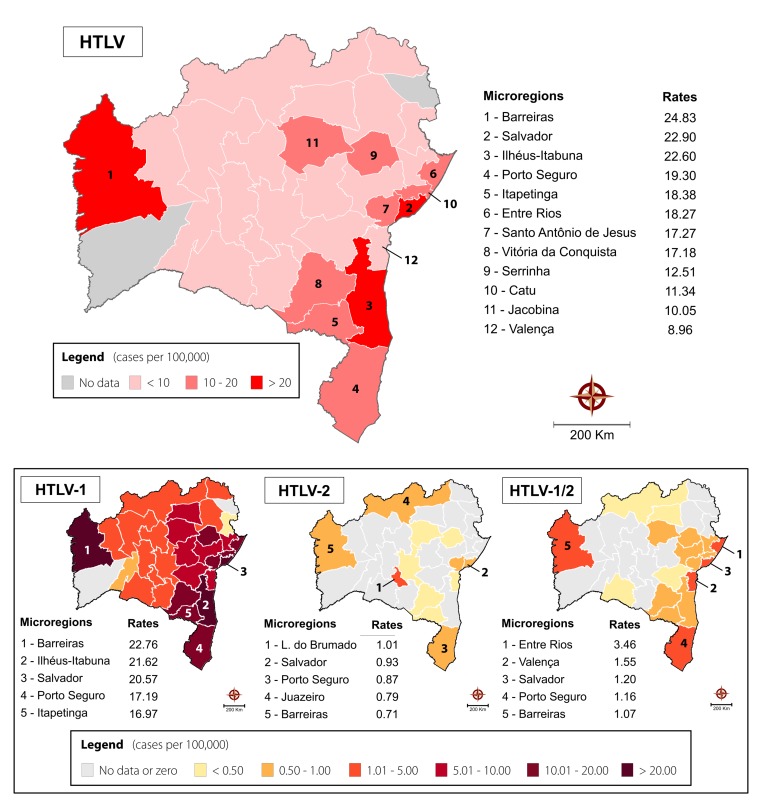
Spatial distribution of overall HTLV, HTLV-1, HTLV-2, and HTLV-1/2 positivity in the State of Bahia from 2004 to 2013, considering microregions as the unit of analysis. The 12 microregions with the highest rates of HTLV positivity per 100,000 inhabitants are highlighted, in addition to the top five according to each respective HTLV type.

The cumulative mean rate of HTLV-positive cases in Bahia was 14.4 per 100,000 inhabitants. Considering the grouping of HTLV-positive samples into microregions (Figure 4), three demonstrated rates above 20 HTLV-positive cases per 100,000 inhabitants: Barreiras (24.83 cases per 100,000 inhabitants), Salvador (22.90 cases per 100,000 inhabitants), and Ilhéus-Itabuna (22.60 cases per 100,000 inhabitants). Elevated rates of HTLV infection ranging from 8.96 to 19.30 cases per 100,000 inhabitants were also seen in nine other microregions. In the remaining microregions, a homogenous distribution of HTLV positivity was observed. No information was retrieved from two microregions (Santa Maria da Vitória and Jeremoabo).

The microregions of Barreiras, Salvador and Porto Seguro were all notable for high rates of HTLV-1 and HTLV-2 infection, as well as HTLV-1/2 coinfection. HTLV-1 was also predominant in Ihéus-Itabuna and Itapetinga microregions. Higher rates of HTLV-2 infection were also found in the Livramento do Brumado and Juazeiro microregions. HTLV-1/2 coinfection was most prevalent in the Entre Rios and Valença microregions (Figure 4).

**FIGURE 5:**
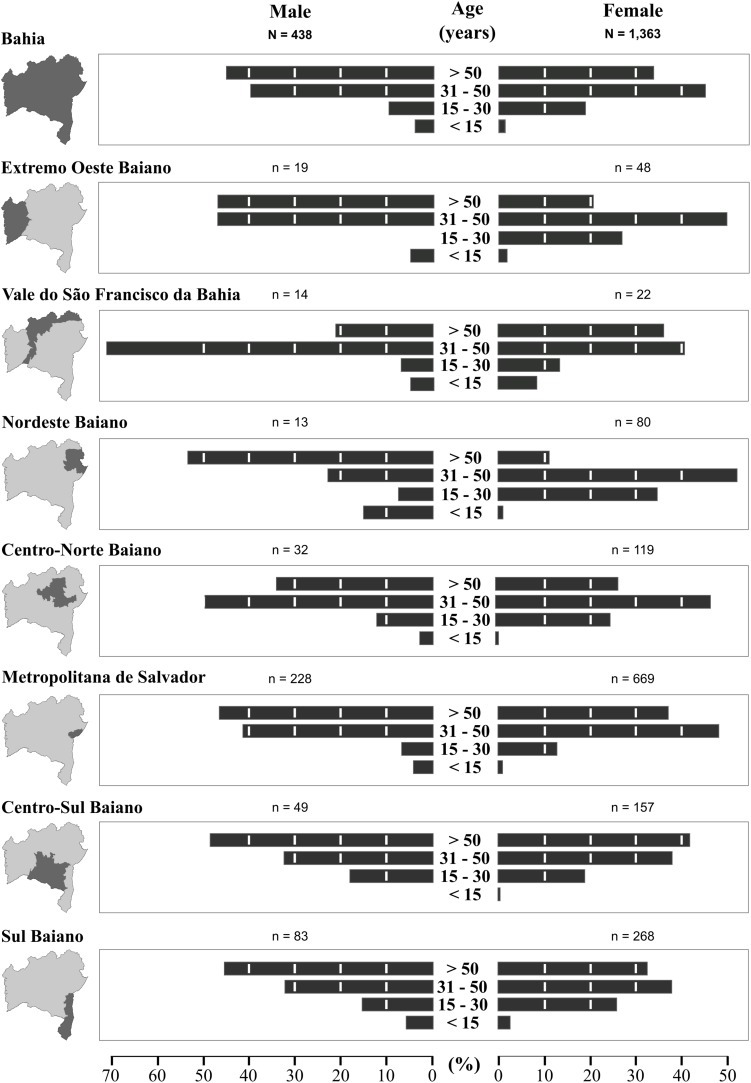
Distribution of HTLV-positive cases throughout the mesoregions of the State of Bahia, stratified according to sex and age (2004–2013).

The median age of the studied population was 31 years (interquartile range: 25–39 years) and the female:male ratio was 8:1. The median age of HTLV-infected persons was 46 years (IQR: 33–59 years), and 75% were women. Overall, our analysis of HTLV-positivity according to age and sex showed much higher frequencies in individuals aged 31 years or older for both males (85.7%) and females (79.3%) (Figure 5). Our stratified age analysis identified 42 positive individuals aged 15 years or younger from all of the mesoregions. The highest frequency of this age group was found in Nordeste Baiano. Three children, two male and one female, younger than 48 months of age, tested positive for HTLV.

In two mesoregions, Extremo Oeste Baiano and Nordeste Baiano, higher frequencies of positive cases were observed among females aged 15–30 years as compared to males. Conversely, a higher frequency of positive cases among males aged >30 years was found in the Vale do São Francisco da Bahia mesoregion.

## Discussion

Salvador, the capital of the Brazilian state of Bahia, has been considered the epicenter of HTLV-1 infection in Brazil. A population-based study conducted in 2003 estimated a total of 40,000 infected individuals in this city (Dourado et al., 2003). Scattered studies have evidenced the presence of the virus in specific population groups throughout the state, such as pregnant women (Bittencourt et al., 2001; Magalhaes et al., 2008; Mello et al., 2014). To date, no information on the number of HTLV-infected individuals in the State of Bahia (the fourth-most populous in Brazil), nor the geographical distribution of these individuals, has been available. The present results demonstrate that HTLV infection is indeed widespread throughout the state, with an overall rate of 14.4 per 100,000 inhabitants. HTLV-1 can be considered predominant, as it corresponds to 91.2% of all cases, followed by HTLV-1/HTLV-2 coinfection (5.4%) and HTLV-2 (2.9%).

Notably, our results identified one important cluster of HTLV infection comprising four microregions surrounding the Salvador microregion (Entre Rios, Santo Antonio de Jesus and Catu). This cluster consists of 43 cities and concentrates almost a third of the state’s population. Geographically, the municipalities in this cluster are mainly located in the area surrounding the Baía de Todos os Santos, which is considered to have a subtropical climate characterized by Atlantic Forest. The population is largely of similar ethnic origin (mainly of African descent) with uniform socialdemographic characteristics. As expected, the Salvador microregion which includes 10 municipalities, presented a high overall rate of HTLV-infection (22.90/100,000 inhabitants). This finding confirms previous reports indicating that Salvador is highly affected by this virus (Dourado et al., 2003). Cruz das Almas, a city located in the Santo Antônio de Jesus microregion was also previously identified as having a 1% prevalence in pregnant women (Magalhaes et al., 2008).

Unexpectedly, the present results also identified three other endemic clusters: one located in the southernmost (comprising four microregions, 86 municipalities, ∼ 2.7 million inhabitants), one in the central region (two microregions, 33 municipalities, ∼750.000 inhabitants) and another in the westernmost region of the state (comprising one microregion, 7 municipalities, ∼280.000 inhabitants). Scarce studies identified the presence of HTLV-1 infection in some cities located in these regions: a recent study evaluating pregnant women in Ilhéus and Itabuna, cities in the southern region of Bahia, found 1.05% were infected with HTLV-1 (Mello et al., 2014), while another study found HTLV-1 infection in people from the Jacobina microregion (Britto et al., 1998; Rego et al., 2008). The Ilhéus-Itabuna microregion is located around the southern coastal region of Bahia, having a humid tropical climate with areas of Atlantic Forest. This regional center is home to important commercial, service, and industrial activities. The economic importance of this region grew during the golden era of cocoa production. Nowadays, the economy is based on tourism and other activities related to the seaport. By contrast, the Jacobina microregion has a tropical climate with a dry season. Surrounding lakes, mountains, rivers and waterfalls favor ecological tourism. Regarding the Barreiras microregion, located in the westernmost part of Bahia, to the best of our knowledge no previous studies reported this area being endemic to HTLV infection. We cannot exclude the possibility that these microregions received samples from surrounding areas. Barreiras is the most important municipality located in the microregion, and the city borders Tocantins state. This microregion has a tropical climate and the predominant vegetation is the arboreal cerrado. The regional economy is mainly based on agribusiness, and migrants from all over Brazil moved here in the 1970s and 1980s.

While higher rates of HTLV-1/2 co-infection were mainly present in some microregions identified as HTLV-1 clusters, including Salvador, Barreiras and Porto Seguro, the highest rates of HTLV-2 were present in Livramento do Brumado microregion, located in the center of the state, and Juazeiro, located in the north. In Brazil, few studies have reported on the prevalence of HTLV-1/2 coinfection (Gabbai et al., 1993). HTLV-2 infection has been predominantly reported in indigenous populations residing in northern Brazil (Ishak et al., 1995) and among injectable drug users in urban areas (Gabbai et al., 1993). It is possible that HTLV-1/2 coinfection found in Bahia may result from the introduction of drug users infected with HTLV-2 into areas in which HTLV-1 is already prevalent.

Of note, a progressive increase in the incidence of HTLV infection was observed throughout the study period. This is likely due to an expansion in the number of municipal primary health clinics provided by the federal Family Health Program, as well as increases in testing and counseling centers (aimed primarily at sexually transmitted diseases) in the countryside of the state. In addition, awareness surrounding HTLV infection increased during the study period and HTLV was included on the list of compulsory disease notifications for the state of Bahia in 2011.

With respect to HTLV infected individuals profile, the mean age was 46 years with a predominance of female (75%). HTLV infection is known to be more prevalent in women, and its prevalence increases with age (Dourado et al., 2003; Mota et al., 2006). Herein, a higher proportion of HTLV-1-infected women was detected in the 31–50 age range among most mesoregions. This could be due to the non-random nature of the sample, which was young (median age of 31 years) and consisted mostly of females. Increased sexual activity is more common in this age range, and virus transmission is known to be more efficient from men to women (Murphy et al., 1989). It is also possible that compulsory serological HTLV screening for pregnant women, in effect in Bahia, contributed to the higher proportion of women detected in this age range.

By contrast, in the Vale do São Francisco and Centro Norte Baiano mesoregions, men were found to be predominantly infected by HTLV in the age range of 31–50 years. Both mesoregions are highly productive agricultural zones containing municipalities of high population density. In addition, the Centro Norte Baiano mesoregion attracts young men to work in the mining industry. Accordingly, it is theoretically possible that the higher prevalence observed in men of this age range could be due to increased exposure to illicit drugs, as well as to an increased incidence of STDs, such as HIV, gonorrhea and syphilis. In addition, lower levels of formal education and income have been attributed to a higher risk of acquiring HTLV infection (Dourado et al., 2003). Moreover, despite the greater efficiency of male-female transmission, evidence of female-male transmission has also been reported (Brodine et al., 1992). Concerning the possible route of the HTLV transmission, it has been suggested that in Salvador the sexual route predominates, since almost no children was found to be infected (Dourado et al., 2003; Nunes et al., 2017). In the present study the HTLV infection was observed in 42 individuals under 15 years, indicating that in addition to sexual transmission, the vertical route may be an important route.

**FIGURE 6:**
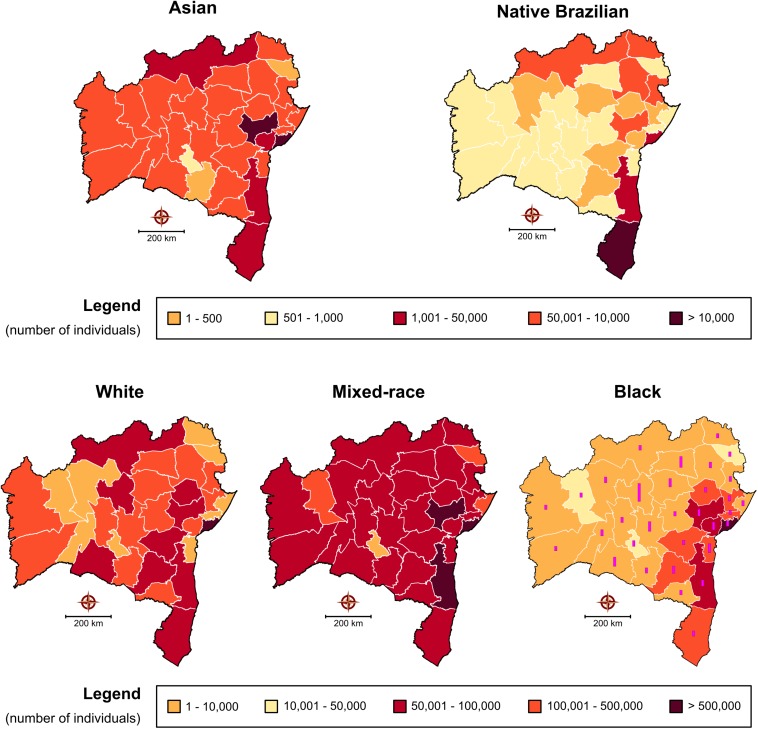
Self-reported ethnicity/color of regional populations in the state of Bahia according to the Brazilian Institute of Statistics and Geography 2010 census. With respect to the representative map of individual identifying as “black,” bars represent the number of quilombola communities located in each mesoregion of the state (total number = 656).

Brazil is considered an endemic area for HTLV (Gessain and Cassar, 2012). However, the prevalence of this infection in the country varies according to the geographic region, being higher in the North and Northeast (Galvao-Castro et al., 1997). The origin of HTLV in Brazil is linked to the introduction of the virus in the post-Colombian era, by the slave trade from Africa mainly to the northeastern cities of the country (Alcantara et al., 2003; Magalhaes et al., 2008; Rego et al., 2008). The population in Bahia is highly mixed, consisting of mostly black and mixed Western African and Portuguese descendants (Azevedo et al., 1982). Figure 6 illustrates the distribution in each microregion of self-reported ethnicity/color (white, mixed-race, black, Asian, or native Brazilian) according to the data reported by the Brazilian Institute for Geography and Statistics (IBGE) in its 2010 census^3^. A diffuse distribution is observed throughout the microregions, especially with respect to white/mixed-race and black, which corresponds to the distribution of HTLV-1 cases, while native Brazilians are more concentrated in the Porto Seguro microregion, which also had a notable rate of HTLV-2 infection. However, the information available prevents us from making direct correlations between ethnic profile and the distribution of HTLV types. Interestingly, the data herein indicate that the microregions with the highest rates of infection in the state of Bahia were areas where communities recognized as quilombos predominated. Originally, quilombos were places of refuge for the African and Afro-descendant slaves who fled slavery. Their descendants often remained in these areas and were then termed quilombolas. Bahia is one of the states with the highest number of quilombolas recognized^4^, Figure 6 (Black).

One limitation of the present study was the non-random sample, which was predominantly female. However, our analysis considered samples from 94% (394/417) of the municipalities in Bahia, a state with an estimated population of 15,126,371 inhabitants; i.e., just 5.5% of the municipalities (comprising a total of 260,125 inhabitants) did not send any samples to LACEN during the study period. This was especially relevant in two microregions (Santa Maria da Vitória and Jeremoabo), from which no information regarding HTLV testing was obtained. To explain this, we can only speculate that perhaps local health authorities chose to outsource testing to private laboratory services instead of sending samples to LACEN, or that authorities in these municipalities did not properly implement actions designed to increase HTLV detection. In fact, the municipality of Jeremoabo has, to date, not reported a single case of HTLV to the Information System on Diseases of Compulsory Declaration (Sistema de Informação de Agravos de Notificação—Sinan), while Santa Maria da Vitória reported a single case in 2013 and another in 2018 (data not shown). Accordingly, we surmise that health professionals in these areas faced challenges in identifying patients suspected of HTLV infection, and/or did not recognize HTLV infection as a sexually transmitted infection, and therefore did not request appropriate serological testing. While population-based studies are considered the gold standard for estimating infection prevalence, high cost and methodological difficulties can present significant challenges (Hlela et al., 2009). Accordingly, estimates of HTLV prevalence are often based on data obtained from blood banks, pregnant women and other population groups (Verdonck et al., 2007b). In addition, the present study is unable to offer any insight into virus phylogeny, as the retrospective nature of this investigation precluded the performance of molecular analysis to accurately assess results considered indeterminate by Western Blot.

## Conclusion

In conclusion, the present study serves to highlight previously unreported data regarding clusters of HTLV infection throughout the state of Bahia, which is considered to be the Brazilian state with the highest HTLV infection rate. HTLV infection cannot be considered as restricted to the area of Salvador, the capital of Bahia, as the data reported herein identified new endemic areas. Considering the overall prevalence of HTLV in Bahia to be 0.84% (1,978/233,876), it follows that ∼130,000 individuals would be infected with HTLV. However, it is important to note that this number could be underestimated, since 26% of the HTLV reactive samples were not submitted to Western blotting, and were thusly excluded. Had 85.2% of these samples been confirmed, and subsequently included in our analysis, the overall prevalence would increase to 1.18%. Further studies are needed to better describe the epidemiological profile of the infected population and to reinforce public policies designed to prevent HTLV transmission, especially in pregnant women.

## Ethics Statement

As the present study has a retrospective design, evaluating HTLV serologies performed in a 10 years-period in the Public Health Laboratory of Bahia, the Institutional Research Board of Fiocruz approved a study, dispensing the signing of written informed consent.

## Author Contributions

All authors read and approved the final version of the manuscript, analyzed and interpreted the data, and drafted the manuscript. FP, MC, MG, and BC conceived and designed the study. FP, MC, CR, RC, and MG acquired the data. FP, MG, FS, and BC critically revised the manuscript.

## Conflict of Interest Statement

The authors declare that the research was conducted in the absence of any commercial or financial relationships that could be construed as a potential conflict of interest.

## Footnotes

1 http://www.ibge.gov.br

2 www.dpi.inpe.br/terraview

3 http://www.ibge.gov.br

4 http://www.palmares.gov.br/?page_id=37551
